# P-1371. Real-World Performance of the "TB or Not TB" Tuberculosis Diagnostic Clinical Decision Support System

**DOI:** 10.1093/ofid/ofaf695.1558

**Published:** 2026-01-11

**Authors:** Caitlin Dugdale, Kimon C Zachary, Lindsay Germaine, Chloe V Green, Rocio M Hurtado, Emily P Hyle, Michelle S Jerry, Jacob E Lazarus, Stephen Maxfield, Molly Paras, Katherine Swanson, Erica S Shenoy

**Affiliations:** Massachusetts General Hospital, Boston, MA; Massachusetts General Hospital, Boston, MA; Mass General Brigham, Somerville, Massachusetts; Massachusetts General Hospital, Boston, MA; Massachusetts General Hospital, Boston, MA; Massachusetts General Hospital, Boston, MA; Massachusetts General Hospital, Boston, MA; Massachusetts General Hospital, Boston, MA; Massachusetts General Hospital, Boston, MA; Massachusetts General Hospital; Massachusetts General Hospital, Boston, MA; Mass General Brigham, Somerville, Massachusetts

## Abstract

**Background:**

We implemented a validated clinical decision support system (CDSS) to guide clinicians through the diagnostic evaluation of individuals with suspected active tuberculosis across the Mass General Brigham System (MGB) in August 2024. TBorNotTB assigns points based on epidemiologic risk factors, tuberculosis history, symptoms, chest imaging, and sputum/bronchoscopy results. If the CDSS score is < 1, the evaluation is complete and airborne infection isolation (AII) precautions are discontinued; otherwise, additional evaluation is recommended, including review by infection control personnel. Our objective was to assess early real-world CDSS performance.

**Methods:**

We conducted a prospective study of MGB patients for whom the TBorNotTB CDSS was used from August 2024-January 2025. We evaluated the frequency of CDSS use and the sensitivity, specificity, and the area under the curve (AUC) of the CDSS to detect active tuberculosis (with AII continuation) in comparison to mycobacterial culture and PCR when available. Details of individuals with confirmed tuberculosis were also collected.

**Results:**

Providers used the CDSS for 291 (41%) of the 715 individuals marked in the electronic health record as having suspected tuberculosis. Of these, 4/291 (1.4%) had confirmed tuberculosis and 248/291 (85%) tested negative for tuberculosis by PCR and/or mycobacterial culture (Figure 1). An additional 39/291 (13%) did not have mycobacterial culture or PCR performed. Among uses of the CDSS in which there was corresponding mycobacterial culture or PCR available (N=252), the sensitivity and specificity of the tool to detect tuberculosis were 100% and 60%, respectively. Median CDSS scores for individuals with and without TB were 8 (range: 2-19) and 0 (range: -12-18), respectively (Figure 2), yielding an AUC of 0.84. Individuals with confirmed tuberculosis are described in Table 1.

**Conclusion:**

With real-world use, the TBorNotTB CDSS demonstrated 100% sensitivity and moderate specificity to predict tuberculosis among individuals with suspected tuberculosis in a low-prevalence setting. This CDSS could help reduce risks of nosocomial tuberculosis transmission and infection control person-time spent reviewing individuals with suspected tuberculosis.

**Disclosures:**

Erica S. Shenoy, MD, PhD, Up to Date: Author

